# Inhibition of Panx1/P2X7R/NLRP3 Signaling Attenuates Bone Cancer Pain and Spinal Microglial Activation

**DOI:** 10.3390/biomedicines14102183

**Published:** 2026-09-26

**Authors:** Ying Liang, Jianshen Song, Chenchen Wang, Yue Qian, Rui Xu, Yulin Huang, Bailing Hou, Yanting Mao, Zhengliang Ma

**Affiliations:** Department of Anesthesiology, Nanjing Drum Tower Hospital, The Affiliated Hospital of Medical School, Nanjing University, Nanjing 210008, China; abalaying@hotmail.com (Y.L.); doctorsong92@163.com (J.S.); m15805165976_1@163.com (C.W.); qianyue@stu.njmu.edu.cn (Y.Q.); salt0226@163.com (R.X.); hyl199006@126.com (Y.H.)

**Keywords:** bone cancer pain, spinal microglia, Panx1, P2X7R, NLRP3

## Abstract

**Objectives**: This study aims to investigate whether regulation of the Panx1/P2X7R/NLRP3 signaling axis in spinal microglia alleviates bone cancer pain. **Methods**: An in vivo bone cancer pain model was established by inoculating osteolytic tumor cells into the femoral medullary cavity of C3He/J mice. Nociceptive behaviors were evaluated using paw withdrawal mechanical thresholds and spontaneous flinch counts. The expression levels of Panx1, P2X7R, and the NLRP3 in the spinal dorsal horn (SDH) were examined. Analgesic effects of intrathecally administered ^10^Panx, BBG, and MCC950 were evaluated, and the intracellular localization of Panx1 was determined using immunofluorescence. In Panx1-knockdown BV2 cells, ATP-induced activation of the P2X7R-Panx1 pathway, IL-1β and IL-18 production, and ROS accumulation were assessed. **Results**: Tumor inoculation induced mechanical allodynia and increased spontaneous flinches. Moreover, Panx1, P2X7R, and NLRP3 were upregulated in the mouse SDH, accompanied by increased levels of caspase1, IL-1β, and IL-18. Intrathecal administration of ^10^Panx, BBG, and MCC950 significantly alleviated bone cancer pain related behaviors in mice. Panx1 was predominantly localized in activated microglia of the SDH following tumor inoculation. In ATP-stimulated BV2 cells, Panx1 knockdown markedly attenuated P2X7R and NLRP3 expression, reduced IL-1β and IL-18 production, and diminished intracellular ROS accumulation. **Conclusions**: Tumor cell inoculation promotes the release of pro-inflammatory cytokines and enhances nociceptive transmission by activating the Panx1/P2X7R/NLRP3 signaling axis in the spinal microglia, thereby contributing to the development of bone cancer pain. Targeting the Panx1/P2X7R/NLRP3 signaling axis may therefore represent a promising therapeutic strategy for treating bone cancer pain.

## 1. Introduction

Bone cancer pain (BCP), a treatment-resistant form of pain prevalent among cancer patients, is caused by bone metastasis and destruction, which are more common in individuals with metastatic breast, lung, and prostate cancer [1]. Statistics show that approximately 75% patients with advanced cancer experience moderate-to-severe BCP [2]. Moreover, over 50% of patients with metastatic cancer pain report insufficient pain relief with current therapeutic pharmacotherapies (e.g., opioids and nonsteroidal anti-inflammatory drugs) due to their limited efficacy and potential for addiction [3,4]. Therefore, there is an urgent need to design novel analgesic modalities to improve the palliative care, pain relief, and quality of life in cancer patients suffering from BCP.

The pathogenesis of BCP involves a complex interplay among tumor cells, bone destruction, peripheral sensory neurons, and the central nervous system. Among these mechanisms, spinal neuroinflammation has emerged as a critical contributor to the initiation and maintenance of chronic pain [5]. Microglia-mediated neuroinflammation contributes substantially to refractory chronic pain and represents a promising therapeutic target [6]. Microglia sensitively and specifically sense graded changes in neuronal excitability [7,8]. In sustained pain states, spinal dorsal horn microglia actively monitor fluctuations in extracellular neurotransmitter concentrations (e.g., glutamate, glycine, and ATP), playing an integral role in nociceptive signal transduction during chronic pain progression [9,10]. Mounting evidence indicates that a passive neuroimmune cascade—initiated by bone tumor progression, aggravated by pro-inflammatory mediator release and microglial activation, and culminating in hyperalgesia following neural injury—plays a dominant role in BCP development [11,12,13]. Persistent microglial activation in the SDH significantly affects the temporal dynamics and morphological plasticity of BCP progression [14], during which distinct neuroelectrophysiological alterations arise [15].

Pannexins form large-pore channels that are permeable to ATP and other signaling molecules. Pannexin-1 (Panx1), the most typical member of the pannexin family, is ubiquitously expressed in neurons and glial cells of the nervous system [15,16]. Activated Panx1 in the spinal cord, astrocytes, and microglia participates in pain development, accompanied by morphological activation of microglia in the SDH [17,18]. Mechanistically, this process is critically driven by Panx1 channel opening and NLRP3 inflammasome (NOD-like receptor family pyrin domain containing 3, NLRP3) activation, which jointly facilitate inflammatory signal release [19]. Specifically, NLRP3 inflammasome activation is triggered by the interaction of Panx1 channels with ATP-sensitive P2X7 purinoceptors or by the promotion of potassium efflux. This activation leads to the release of pro-inflammatory factors (e.g., IL-1β and IL-18), which in turn trigger, exacerbate, and sustain the inflammatory response [20]. Whether Panx1-mediated activation of spinal microglia drives NLRP3 inflammasome activation during BCP, and whether pharmacological inhibition of this signaling cascade alleviates pain hypersensitivity, have not been fully established.

In the present study, we investigated the activation of the Panx1/P2X7R/NLRP3 signaling axis in the spinal dorsal horn using a mouse model of bone cancer pain. We further evaluated the analgesic effects of pharmacological inhibition of Panx1, P2X7R, and NLRP3 in vivo and examined the underlying molecular mechanisms in ATP-stimulated BV2 microglial cells. Our findings demonstrate that the Panx1/P2X7R/NLRP3 signaling is activated in spinal microglia and promotes pro-inflammatory cytokine release in spinal microglia, thereby contributing to the development of bone cancer pain. These results identify the Panx1/P2X7R/NLRP3 signaling axis as a potential therapeutic target for BCP.

## 2. Materials and Methods

### 2.1. Animals

Male C3He/J mice at aged 4–5 weeks and 17–21 g of weight (Vital River Laboratory Animal Technology Co., Ltd. Beijing, China) were housed with 5–6 mice per cage, under a 12-h light/dark cycle with ad libitum access to food and water. They were acclimatized for at least one week prior to experimental procedures. All experiments were conducted in strict accordance with the guidelines approved by the Experimental Animal Ethics Committee of Nanjing Drum Tower Hospital. A total of 227 mice were used in this study, and the number of animals included in each experiment is specified in the corresponding figure legends.

### 2.2. BCP Modeling In Vivo

An in vivo BCP model was created as previously reported [21]. After 1.5–2.0% isoflurane anesthesia via an animal anesthesia machine (Midmark VMR, model 91805060, Versailles, OH, USA) at a maintenance flow rate of 0.2 L/min, the mouse right knee joint was surgically exposed to allow an injection of 20 µL of α-minimum essential medium (Gibco, 12571071, Grand Island, NY, USA) containing 2 × 10^5^ NCTC 2472 murine fibrosarcoma cells into the medullary cavity of the femur. Sham-operated mice received an equal volume of culture medium without tumor cells. Mice were then placed on a warming pad for postoperative recovery.

### 2.3. Micro-Computed Tomography (Micro-CT) Imaging

Bone destruction was evaluated using micro-computed tomography (micro-CT) on postoperative day 21. Femurs from tumor-inoculated or Sham mice were scanned using a Skyscan 1276 scanner (60 kV, 200 µA, 0.5 mm Al filter, 20 µm pixel size; Bruker, Kontich, Belgium). Reconstruction and position correction were performed using Nrecon v2.0.0.5 and DataViewer v1.5.6.2. Bone destruction was scored on a 0–5 scale (0: normal; 1: 1–3 radiolucent lesions; 2: 3–6 lesions with medullary bone loss; 3: cortical erosion; 4: unicortical bone loss; 5: bicortical loss with fractures) [22]. Trabecular parameters were quantified using a CT Analyzer v1.17.7.2, with a 3D visualization via CTVox v3.3.0. Investigators who were responsible for micro-CT imaging were blinded to experimental grouping.

### 2.4. Pharmacological Intervention

To investigate the contribution of the Panx1/P2X7R/NLRP3 signaling pathway to BCP, mice were given a single intrathecal injection of NLRP3 inflammasome inhibitor MCC950 (T3701, Sigma Aldrich, St. Louis, MO, USA), Panx1 channel inhibitor ^10^Panx (HY-P1137, MCE, Shanghai, China), or P2X7 receptor antagonist Brilliant Blue G (BBG, HY-D0014, MCE, China) in a total volume of 5 µL. Briefly, mice in the Tumor + MCC950 group were intrathecally injected with 10, 20, and 40 µg MCC950 dissolved in sterile saline, and those in the Tumor + ^10^Panx group were similarly given 0.31 μg, 0.62 μg, and 1.24 μg ^10^Panx dissolved in sterile saline containing 10% dimethyl sulfoxide (DMSO). Mice in the Tumor + BBG group were treated with a single intrathecal injection of 0.03, 0.06, and 0.12 µg BBG. The same volume of 10% DMSO in saline or sterile saline was administered to mice in the Tumor + Vehicle group. Drugs were administered on postoperative day 14.

### 2.5. Nociceptive Behavior Test

Mechanical hypersensitivity and spontaneous nocifensive behavior were detected via measurements of paw withdrawal mechanical threshold (PWMT) and the number of spontaneous flinches (NSF) on the day prior to surgery (baseline, BL) and on postoperative days 4, 7, 10, 14, and 21, with 8 mice per group. Behavioral assessments were performed by investigators blinded to treatment allocation.

PWMT. PWMT was measured using a series of von Frey filaments weighing 0.16, 0.4, 0.6, 1.0, 1.4, and 2.0 g (Stoelting Co., Wood Dale, IL, USA), and mechanical allodynia was evaluated via the up–down method as previously reported [23]. Before testing, mice were acclimated for at least 30 min in transparent plexiglass compartments with a wire mesh floor. After perpendicularly applying the von Frey filaments to the right plantar, the lowest filament stimulus strength leading to paw flinching or withdrawal was recorded. The PWMT was defined as the lowest filament intensity for eliciting a minimum of three positive responses out of five repeated trials, with a 5-min interval between the two trials. A double-blind design and randomized grouping were used to ensure the rigor of PWMT measurement.

NSF. Spontaneous nocifensive behavior was quantified by counting the NSF of the right hind paw. After a minimum of 30-min acclimation in a clear plexiglas compartment, paw flinching during a 2 min period of each mouse was observed for a total of five trials.

### 2.6. Hematoxylin and Eosin (H&E) Staining

Mouse femoral tissue pre-processing comprised 24 h fixation in 10% paraformaldehyde and a 14-day decalcification in EDTA, followed by dehydration, paraffin embedding, and sliding into 5 μm thick slices. Sections were then stained with hematoxylin and eosin (H&E) using a standard protocol. After hematoxylin staining, the slices were dehydrated in an alcohol gradient, counterstained with eosin, sealed, and captured under a light microscope (Zeiss, Karlsruhe, Germany). A double-blind and randomized design was utilized in spinal cord tissue sampling and pre-treatment and subsequent testing and analysis.

### 2.7. Immunofluorescence Staining

On postoperative day 21, 6 randomly selected mice in the Sham and Tumor groups were anesthetized with 2% isoflurane and perfused transcardially with normal saline and 4% paraformaldehyde. The lumbosacral enlargement was dissected, fixed in 4% paraformaldehyde, and cryoprotected in 30% sucrose at 4 °C. Transverse 20 μm-thick sections of the lumbar spinal cord (L3–L5) were blocked for 1 h in 10% goat serum with 0.3% Triton X-100 and incubated overnight at 4 °C with the mouse anti-Iba1 (1:500, NCNP24, WAKO, Osaka, Japan), rabbit anti-P2X7R (1:100, 282071-AP, Proteintech, Wuhan, China), and rabbit anti-Panx1 antibodies (1:200, 600-401-DJ7, Thermo Fisher, Waltham, MA, USA). They were further incubated with the Alexa 488-conjugated goat anti-rabbit (1:1000, Invitrogen, Waltham, MA, USA, A32731) and Alexa 594-conjugated goat anti-mouse (1:1000, Invitrogen, A11032, Waltham, MA, USA) at room temperature for 1 h, kept in darkness, and stained with 4′,6-diamidino-2-phenylindole (DAPI) (Beyotime, Shanghai, China, P0131). Confocal imaging was captured under a laser-scanning confocal microscope (Olympus Corporation, Tokyo, Japan) and quantified for staining density using ImageJ software (v.2.3.0, National Institutes of Health).

### 2.8. Western Blotting

L3-L5 spinal cord segments obtained from 3 randomly selected mice per group were homogenized in M-PER™ mammalian protein extraction reagent (Thermo Fisher, 78501, Waltham, MA, USA) supplemented with a complete protease and phosphatase inhibitor cocktail and prepared for tissue lysates. Processing comprised 30 min incubation on ice and 15 min centrifugation at 12,000× *g* and 4 °C. The supernatant was collected to quantify protein concentrations using a BCA Protein Assay kit (Beyotime, P0009, Shanghai, China). Following separation using 10% sodium dodecyl sulfate–polyacrylamide gel electrophoresis (SDS-PAGE), samples were electrotransferred onto a polyvinylidene fluoride (PVDF) membrane and immersed for 1 h in 5% non-fat dry milk at room temperature. Membranes were incubated overnight at 4 °C with the rabbit anti-P2X7R (1:1000, 282071-AP, Proteintech, Wuhan, China), rabbit anti-NLRP3 (1:1000, 15101, Cell Signaling Technology (CST), Danvers, MA, USA), rabbit anti-Panx1 (1:1000, 600-401-DJ7, Thermo Fisher, Waltham, MA, USA), rabbit anti-pro-caspase1 (1:1000, ab179515, Abcam, Cambridge, UK), rabbit anti-caspase1 (1:1000, 89332, Cell Signaling Technology (CST), Danvers, MA, USA), rabbit anti-IL-18 (1:1000, ab207323, Abcam, Cambridge, UK), and rabbit anti-IL-1β antibodies (1:1000, ab9722, Abcam, Cambridge, UK). They were then probed with corresponding HRP-conjugated secondary antibodies for 1 h at room temperature. Protein signals were visualized using an enhanced chemiluminescence (ECL) solution, captured with a cooled CCD imaging system (Tanon Science and Technology Co., Ltd. Shanghai, China), and semi-quantified using ImageJ (v.2.3.0, National Institutes of Health).

### 2.9. Cell Culture

NCTC2472 murine fibrosarcoma cells were cultured in NCTC135 medium (Sigma Aldrich, St. Louis, MO, USA) containing 10% horse serum (Gibco, Grand Island, NY, USA) at 37 °C in a 5% CO_2_ incubator (Thermo Fisher, Waltham, MA, USA). BV2 cells were cultured in DMEM (11965092, Gibco, Grand Island, NY, USA) containing 10% fetal bovine serum (FBS) and 100 U/mL penicillin–streptomycin (15070063, Gibco, Grand Island, NY, USA) at 37 °C in a 5% CO_2_ incubator. 

### 2.10. ROS Measurement

Intracellular reactive oxygen species (ROS) generation was determined using the fluorescent probe DCFH-DA (S0033S, Beyotime, Beijing, China). BV2 cells were washed with PBS twice for cell counting. A total of 1 × 10^6^ BV2 cells of indicated groups were incubated at 37 °C with the diluted DCFH-DA at a dose of 10 μM for 30 min. After incubation, cells were washed three times with PBS to remove excess probe. The fluorescence intensity was measured using a multifunctional microplate reader (Spark, Tecan, Mannedorf, Switzerland) by detecting the optical density at λ excitation = 488 nm and λ emission = 525 nm. ROS production was expressed as relative fluorescence intensity normalized to the control group.

### 2.11. ATP Measurement

To quantify ATP levels in the cerebrospinal fluid (CSF), approximately 20 μL of CSF was collected from each mouse under 1.5–2.0% isoflurane anesthesia using a glass capillary tube, with the entire procedure limited to 20 min per animal. On day 21 post-inoculation, CSF samples from both Sham and Tumor groups were assayed using an ATP Assay Kit (Beyotime, S0026, Beijing, China) following the recommended protocol. Briefly, 20 μL of CSF was combined with 100 μL of ATP detection working solution in a black 96-well plate, and the luminescence signal was immediately recorded using a multimode plate reader (Spark Tecan, Mannedorf, Switzerland). ATP concentrations were calculated from a standard curve generated in parallel for each experiment.

### 2.12. Statistical Analysis

Data were presented as mean ± standard deviation. The mice were randomly assigned to experimental groups, and all behavioral, histological, and biochemical analyses were performed by investigators blinded to group allocation whenever applicable. For behavioral data across multiple time points, differences were analyzed using two-way repeated-measures analysis of variance (ANOVA), followed by Bonferroni’s post hoc test for intergroup comparisons at each individual time point. Western blotting data were analyzed using unpaired two-tailed Student’s *t*-tests for two-group comparisons or 2 × 2 factorial ANOVA for four-group comparisons, followed by Bonferroni’s post hoc test if appropriate. All statistical analyses were performed using SPSS 22.0 (IBM Corporation, Armonk, NY, USA) and graphs were formatted using GraphPad Prism Version 9 (GraphPad Software, Boston, MA, USA). A two-sided *p* < 0.05 was considered statistically significant.

## 3. Results

### 3.1. Increased NLRP3 Expression Participates in BCP Progression by Triggering the Release of IL-18 and IL-1β

**Figure 1:**
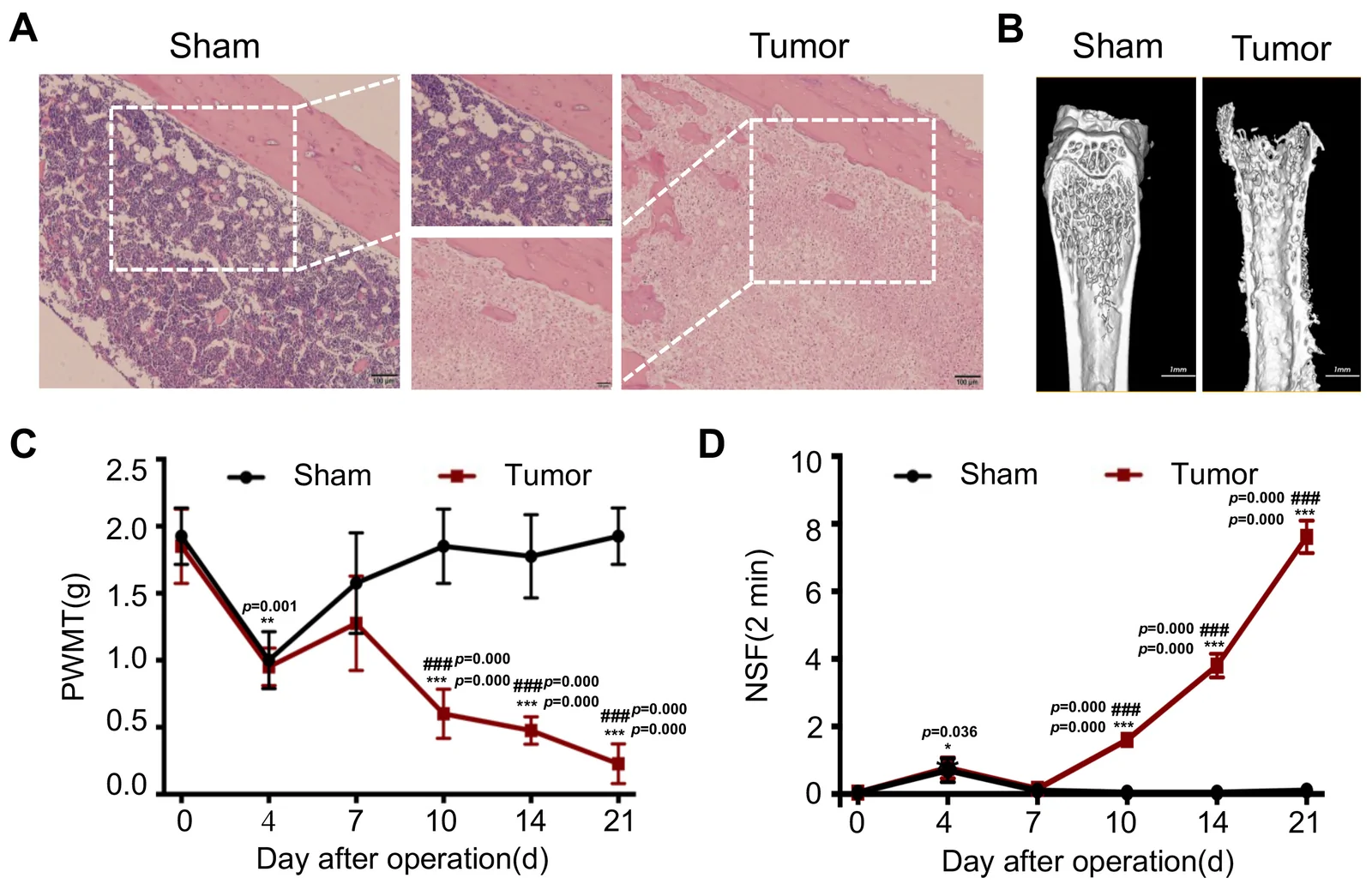
Morphology and behavior changes in Sham and Tumor group mice. (**A**) Representative H&E staining images of tibia sections from Sham and BCP mice at 14 days after NCTC 2472 murine fibrosarcoma cell inoculation. Scale bars = 100 μm and 50 μm. (**B**) Representative Micro-CT images visualizing trabecular and cortical bone destruction in the distal part of tumor-bearing tibia on day 21 after tumor cell inoculation. Scale bar = 1 mm. (**C**,**D**) PWMT (**C**) and NSF (**D**) on postoperative days 0, 4, 7, 10, 14, and 21. *n* = 8. * Compared with day 0, one-way ANOVA using Bonferroni’s post hoc tests. ^#^ Compared with Sham group, repeated-measures ANOVA. PWMT: D0 F(1,14) = 0.368; D4 F(1,14) = 0.304; D7 F(1,14) = 2.695; D10 F(1,14) = 112.179; D14 F(1,14) = 116.187; D21 F(1,14) = 345.127; NSF: D0 F(1,14) = 0.000; D4 F(1,14) = 0.203; D7 F(1,14) = 0.467; D10 F(1,14) = 292.478; D14 F(1,14) = 837.766; D21 F(1,14) = 1786.860.

To validate the successful establishment of the bone cancer pain (BCP) model, histopathological and radiographic examinations were performed. On postoperative day 14 (d14), H&E staining was performed on femoral tissues from mice inoculated with the NCTC 2472 murine fibrosarcoma cell line. Obvious pathological changes were observed in the bone marrow, including tumor cell infiltration within the bone marrow cavity, structural damage to the bone tissue, breakdown of the bone trabeculae, and a loss of osteocytes (Figure 1A). To explore the microarchitecture of the bones, micro-CT with three-dimensional ex vivo reconstruction of the distal portions of tumor-bearing femurs was performed on day 21 after tumor implantation. Notably, pronounced loss of cortical bone was visible compared with Sham (non-tumor-bearing) mice (Figure 1B).

Pain-like behaviors, including spontaneous pain and those evoked by mechanical stress, were evaluated using PWMT and NSF measurements in mice with Tumor and Sham mice. The time course of the evoked mechanical allodynia is shown in Figure 1C. Compared with the Sham group, Tumor group mice exhibited increased pain sensitivity, as evidenced by a significant reduction in the paw withdrawal threshold on days 4, 7, 10, 14, and 21 after tumor cell inoculation (Figure 1C). Similarly, NSF increased significantly from postoperative day 4 onward (Figure 1D), indicating the successful establishment of the BCP model.

**Figure 2:**
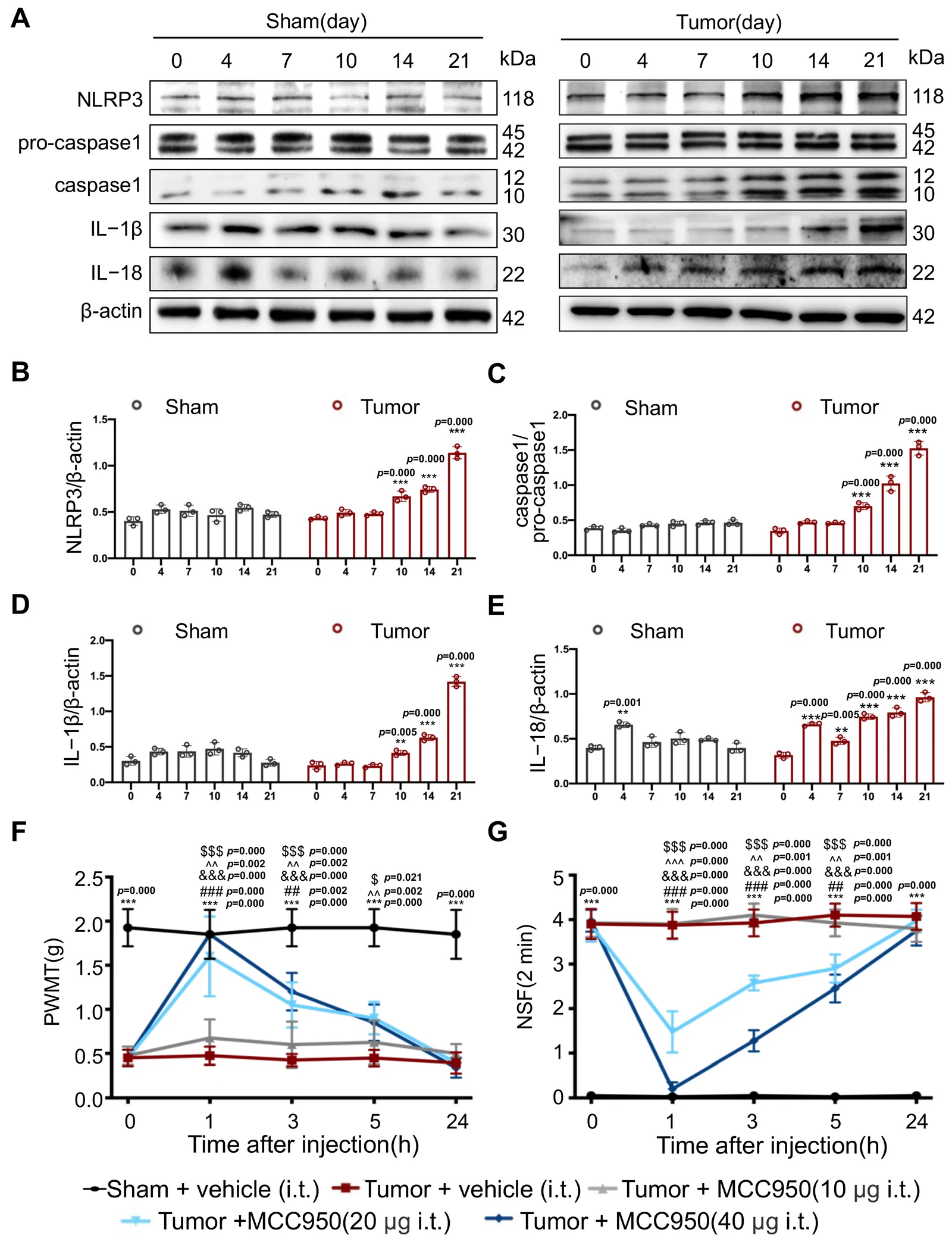
Effects of MCC950 on the releases of IL-18 and IL-1β and BCP progression following NLRP3 signaling activation. (**A**–**E**) Western blotting of NLRP3, pro-caspase1, caspase1, IL-18, and IL-1β on postoperative days 0, 4, 7, 10, 14, and 21 in the Sham and Tumor group, and their quantification analyses (**B**–**E**). *n* = 3. * *p* values were determined using one-way ANOVA followed by the Bonferroni post hoc test. NLRP3: F(5,12) = 123.176; caspase1/pro-caspase1: F(5,12) = 42.509; IL-1β: F(5,12) = 334.129; IL-18: F(5,12) = 105.560. (**F**,**G**) PWMT (**F**) and NSF (**G**) before and after injections of vehicle and 10 μg, 20 μg, and 40 μg MCC950. *n* = 8. *^#&^ Two-way repeated-measures ANOVA followed by the Bonferroni post hoc test was carried out among groups at each time point. * Sham + vehicle vs. Tumor + vehicle, ^#^ Tumor + vehicle vs. Tumor + MCC950 20 μg, ^&^ Tumor + vehicle vs. Tumor + MCC950 40 μg. PWMT: t0: F(4,35) = 211.996; t1: F(4,35) = 44.438; t3:F(4,35) = 60.033; t5: F(4,35) = 68.380; t24: F(4,35) = 130.343. NSF: t0: F(4,35) = 282.815; t1: F(4,35) = 322.440; t3:F(4,35) = 477.239; t5: F(4,35) = 291.896; t24: F(4,35) = 327.401. ^$^^ Paired *t*-tests were performed for efficacy comparisons at each post-dose time point, with Bonferroni’s correction for multiple comparisons. ^^^ Tumor + MCC950 20 μg vs. preinjection, ^$^ Tumor + MCC950 40 μg vs. preinjection.

To further determine the functional significance of NLRP3 activation in mouse SDH, we examined the expression levels of NLRP3 and caspase1/pro-caspase1 in the Sham and Tumor groups. Both NLRP3 and caspase1/pro-caspase1 levels were significantly upregulated on days 10, 14, and 21 compared with the levels before tumor cell inoculation (Figure 2A–C). Elevated expression levels of IL-18 and IL-1β were also observed in the Tumor group, showing trends consistent with increased in NLRP3 expression (Figure 2A,D,E). We further explored the analgesic effect of the NLRP3 inhibitor. On day 14, Tumor group mice received intrathecal injections of MCC950 at doses of 10, 20, or 40 μg. Notably, pain was significantly relieved at doses of 20 and 40 μg as early as 1 h postinjection and this effect lasted until 24 h postinjection (Figure 2F,G). Collectively, these findings indicate that increased NLRP3 expression promotes BCP progression by triggering IL-18 and IL-1β release in the SDH.

### 3.2. BCP Is Associated with Increased Expressions of Microglia P2X7R-Panx1 in the Mouse SDH

**Figure 3:**
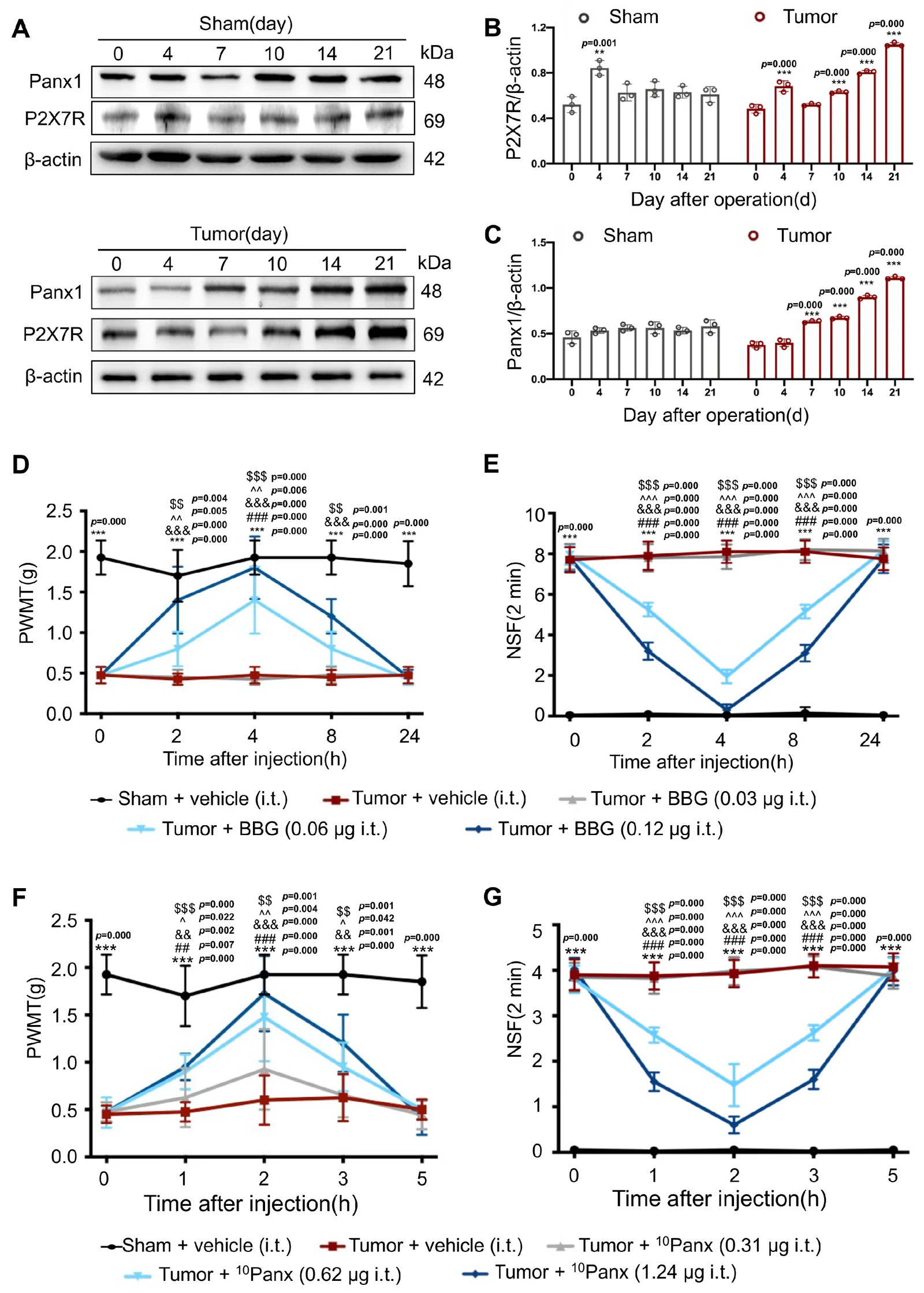
BCP induces the activation of the P2X7R-Panx1 axis. (**A**–**C**) Western blotting of Panx1 and P2X7R on postoperative days 0, 4, 7, 10, 14, and 21 in Sham and Tumor mice (**A**) and their quantification analyses (**B**,**C**). *n* = 3. * *p* values were determined using one-way ANOVA, followed by the Bonferroni post hoc test. P2X7R: F(5,12) = 178.594; Panx1: F(5,12) = 365.313. (**D**,**E**) PWMT (**D**) and NSF (**E**) before and after injections of vehicle and 0.03 μg, 0.06 μg, and 0.12 μg BBG. *n* = 8. *^#&^ Two-way repeated-measures ANOVA followed by the Bonferroni post hoc test was carried out among groups at each time point. * Sham + vehicle vs. Tumor + vehicle,^#^ Tumor + vehicle vs. Tumor + BBG 0.06 μg, ^&^ Tumor + vehicle vs. Tumor + BBG 0.12 μg. PWMT: t0: F(4,35) = 191.447; t2: F(4,35) = 39.362; t4:F(4,35) = 53.892; t8: F(4,35) = 138.672; t24: F(4,35) = 138.729. NSF: t0: F(4,35) = 315.411; t2: F(4,35) = 340.213; t4: F(4,35) = 730.752; t8: F(4,35) = 492.379; t24: F(4,35) = 351.319. ^$^^ Paired *t*-tests were performed for efficacy comparisons at each post-dose time point, with Bonferroni’s correction for multiple comparisons. ^^^ Tumor + BBG 0.06 μg vs. preinjection, ^$^ Tumor + BBG 0.12 μg vs. preinjection. (**F**,**G**) PWMT (**F**) and NSF (**G**) before and after injections of vehicle and 0.31, 0.62, and 1.24 μg ^10^Panx. *n* = 8. *^#&^ Two-way repeated-measures ANOVA followed by the Bonferroni post hoc test was carried out among groups at each time point. * Sham + vehicle vs. Tumor + vehicle, ^#^ Tumor + vehicle vs. Tumor + ^10^Panx 0.62 μg, ^&^ Tumor + vehicle vs. Tumor + ^10^Panx 1.24 μg. PWMT: t0: F(4,35) = 169.165; t1: F(4,35) = 33.836; t2: F(4,35) = 18.289; t3: F(4,35) = 35.683; t5: F(4,35) = 98.461. NSF: t0: F(4,35) = 314.734; t1: F(4,35) = 370.842; t2: F(4,35) = 305.631; t3: F(4,35) = 589.019; t5: F(4,35) = 368.538. ^$^^ Paired *t*-tests were performed for efficacy comparisons at each post-dose time point, with Bonferroni’s correction for multiple comparisons. ^^^ Tumor + ^10^Panx 0.61 μg vs. preinjection, ^$^ Tumor + ^10^Panx 1.24 μg vs. preinjection.

Previous studies have shown that the P2X7R-Panx1 axis, a crucial mediator of spinal pain, is positively expressed on microglia [24]. We next investigated its expression during BCP progression. In the present study, protein levels of Panx1 and P2X7R in the SDH of BCP and Sham group mice were measured at the indicated time points. The expression level of Panx1 was significantly upregulated from day 7 and maintained a high level until day 21 in the BCP group (Figure 3A,C). P2X7R was significantly upregulated on postoperative days 4, 10, 14, and 21 in the BCP group compared with the Sham group (Figure 3A,B). These temporal changes suggest progressive activation of the Panx1–P2X7R signaling pathway during BCP development.

To target the spinal P2X7R-Panx1 axis, mice with established BCP received an intrathecal injection of the P2X7R antagonist BBG (0.03, 0.06, and 0.12 μg) or the Panx1 inhibitor ^10^Panx (0.31, 0.62, and 1.24 μg). A single intrathecal injection of BBG at 0.06 μg and 0.12 μg, but not 0.03 μg, produced gradual increases in PWMT and NSF, which peaked at 4 h and returned to preinjection levels at 24 h (Figure 3D,E). Panx1 channel activation, a core consequence of P2X7R activity, was examined in the BCP model. Administration of ^10^Panx caused dose-dependent reversals of PWMT and NSF, peaking at approximately 2 h and returning to baseline by 5 h. However, intrathecal administration of the lowest dose of ^10^Panx (0.31 μg) did not affect either PWMT or NSF in Tumor group mice (Figure 3F,G). Together, these results demonstrate that activation of the P2X7R-Panx1 signaling pathway contributes to bone cancer pain and that pharmacological inhibition of either Panx1 or P2X7R effectively alleviates pain-related behaviors.

### 3.3. Inhibition of P2X7R-Panx1 Alleviates BCP by Suppressing NLRP3 Activation and the Release of IL-1β and IL-18

**Figure 4:**
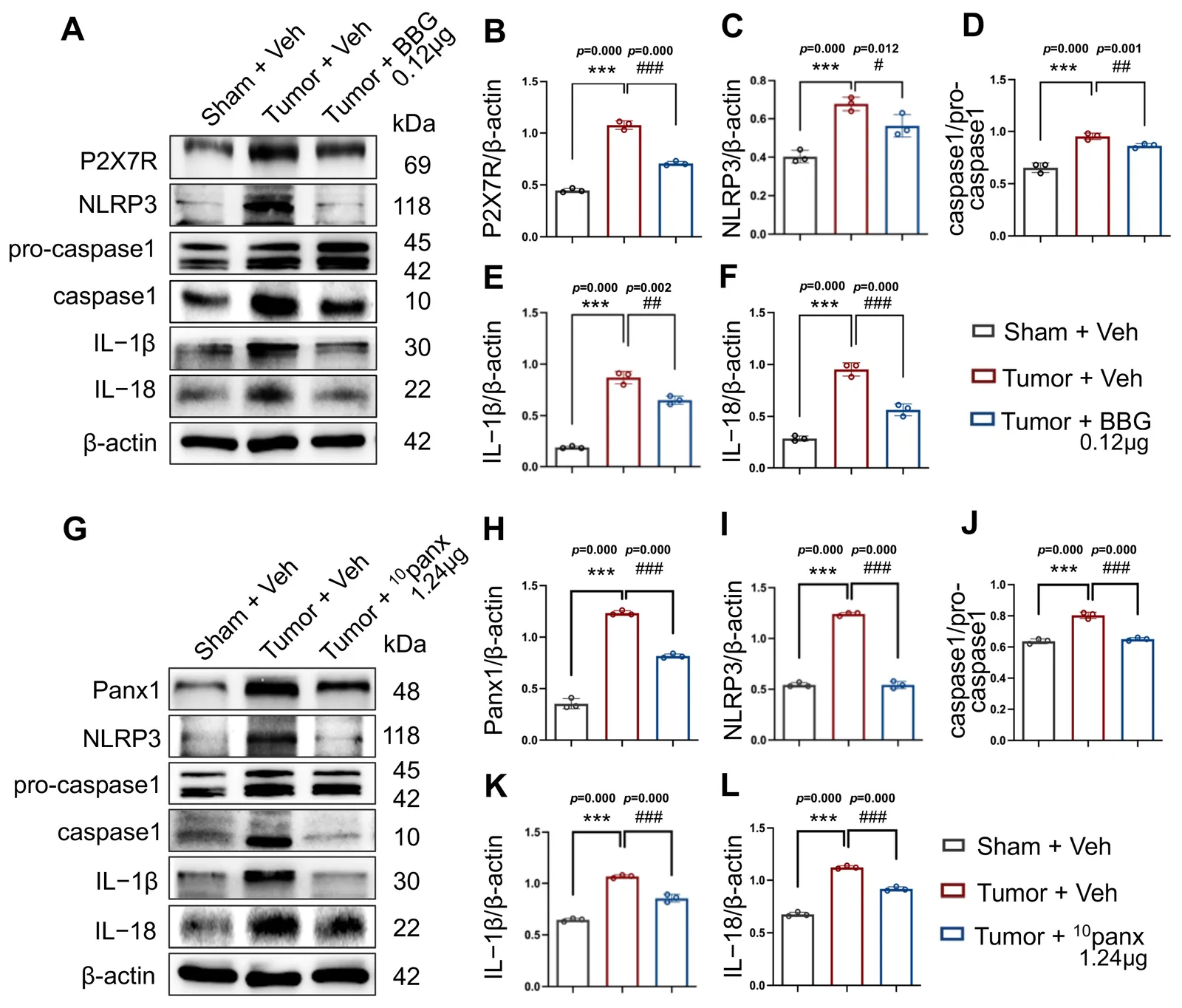
BCP relief achieved by blocking the P2X7R-Panx1 axis to downregulate NLRP3/caspase1/IL-1β/IL-18 in SDH. (**A**–**F**) Western blotting of P2X7R, NLRP3, pro-caspase1, caspase1, IL-18, and IL-1β (**A**) and their quantification analyses (**B**–**F**) in Sham + vehicle, Tumor + vehicle, and Tumor + 0.12 μg BBG mice at 4 h postinjection. *n* = 3. Two-way ANOVA followed by the Bonferroni post hoc test was carried out among groups. P2X7R: F(2,6) = 346.962; NLRP3: F(2,6) = 29.778; caspase1/pro-caspase1: F(2,6) = 60.943; IL-1β: F(2,6) = 201.540; IL-18: F(2,6) = 129.000. * Sham + vehicle vs. Tumor + vehicle, ^#^ Tumor + vehicle vs. Tumor + 0.12 μg BBG. (**G**–**L**) Western blotting of Panx1, NLRP3, pro-caspase1, caspase1, IL-18, and IL-1β (**G**) and their quantification analyses (**H**–**L**) in Sham + vehicle, Tumor + vehicle, and Tumor + 1.24 μg ^10^Panx mice at 2 h postinjection. *n* = 3. Two-way ANOVA followed by the Bonferroni post hoc test was carried out among groups. Panx1: F(2,6) = 512.951; NLRP3: F(2,6) = 704.532; caspase1/pro-caspase1: F(2,6) = 100.696; IL-1β: F(2,6) = 233.471; IL-18: F(2,6) = 408.848. * Sham + vehicle vs. Tumor + vehicle, ^#^ Tumor + vehicle vs. Tumor + 1.24 μg ^10^Panx.

Mounting evidence indicates that P2X7R-Panx1 signaling regulates inflammatory responses relying on the NLRP3 inflammasome [25,26,27]. To assess NLRP3 signaling activation after pharmacological inhibition of P2X7R-Panx1, we compared caspase1/pro-caspase1 and NLRP3 expression in the mouse SDH. Expression levels of P2X7R, Panx1, caspase1/pro-caspase1, and NLRP3 were significantly higher in the Tumor + Vehicle group than in the Sham + Vehicle group (Figure 4A,G). Pharmacological inhibition of P2X7R-Panx1 in the SDH effectively downregulated caspase1/pro-caspase1, and decreased NLRP3 expression (Figure 4A–D,G–J). Consistent with these changes, both BBG and ^10^Panx significantly reduced spinal IL-1β and IL-18 expression compared with vehicle-treated tumor-bearing mice (Figure 4A,E–G,K,L). Taken together, these findings are consistent with the hypothesis that activation of the Panx1–P2X7R signaling pathway may promote NLRP3 signaling activation and production of IL-1β and IL-18 in the spinal dorsal horn during bone cancer pain.

### 3.4. Inhibition of Panx1 Reduces ATP-Induced ROS Accumulation in Microglia

**Figure 5:**
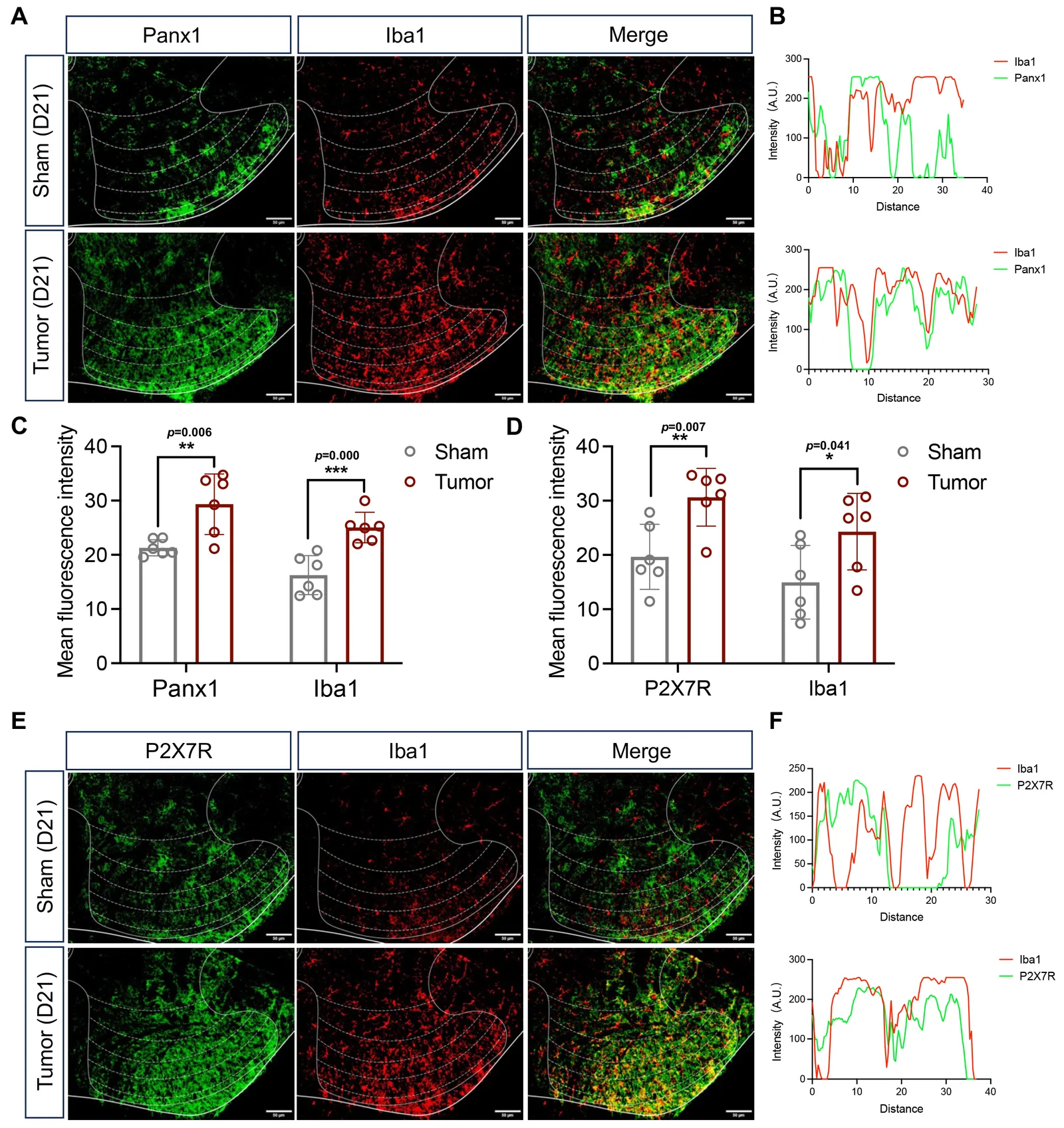
Microglia P2X7R-Panx1 increases in SDH of Tumor group mice. (**A**) Representative immunofluorescence double-staining of Panx1 (green) and Iba1 (red) in SDH of Sham and Tumor groups. Dashed lines indicate the boundaries of the different laminae of the spinal dorsal horn. Scale bar = 50 µm. *n* = 6/group (**B**) Co-localization analysis of Panx1 and Iba1. (**C**,**D**) Quantification of mean fluorescence intensity of Panx1, P2X7R, and Iba1. (**E**) Representative immunofluorescence double-staining of P2X7R (green) and Iba-1 (red) in SDH of Sham and Tumor groups. Scale bar = 50 µm. *n* = 6/group (**F**) Co-localization analysis of P2X7R and Iba1. Comparisons between the two groups were performed using two-tailed unpaired *t*-tests. * Sham vs. Tumor.

To highlight the involvement of microglia in Panx1-mediated mechanical allodynia, microglial expression of Panx1 and P2X7R was analyzed using double immunostaining in the SDH of the Sham and Tumor groups, respectively. The intensities of Panx1 and P2X7R significantly increased in the SDH of the Tumor group mice than in that of Sham group mice (Figure 5A,C–E). Co-localization analysis showed that both Panx1 and P2X7R were highly co-localized with Iba1 in the SDH of Tumor group mice (Figure 5B,F). Quantitative co-localization analysis using non-parametric statistics further supported these observations. Spearman’s rank correlation coefficients were 0.723 ± 0.026 for Panx1/Iba1 and 0.817 ± 0.041 for P2X7R/Iba1, with corresponding Manders’ M1/M2 values of 0.995 ± 0.011/0.995 ± 0.010 and 0.999 ± 0.001/0.994 ± 0.010, respectively. Panx1 was predominantly expressed on microglia and upregulated during the development of mechanical allodynia in BCP mice.

**Figure 6:**
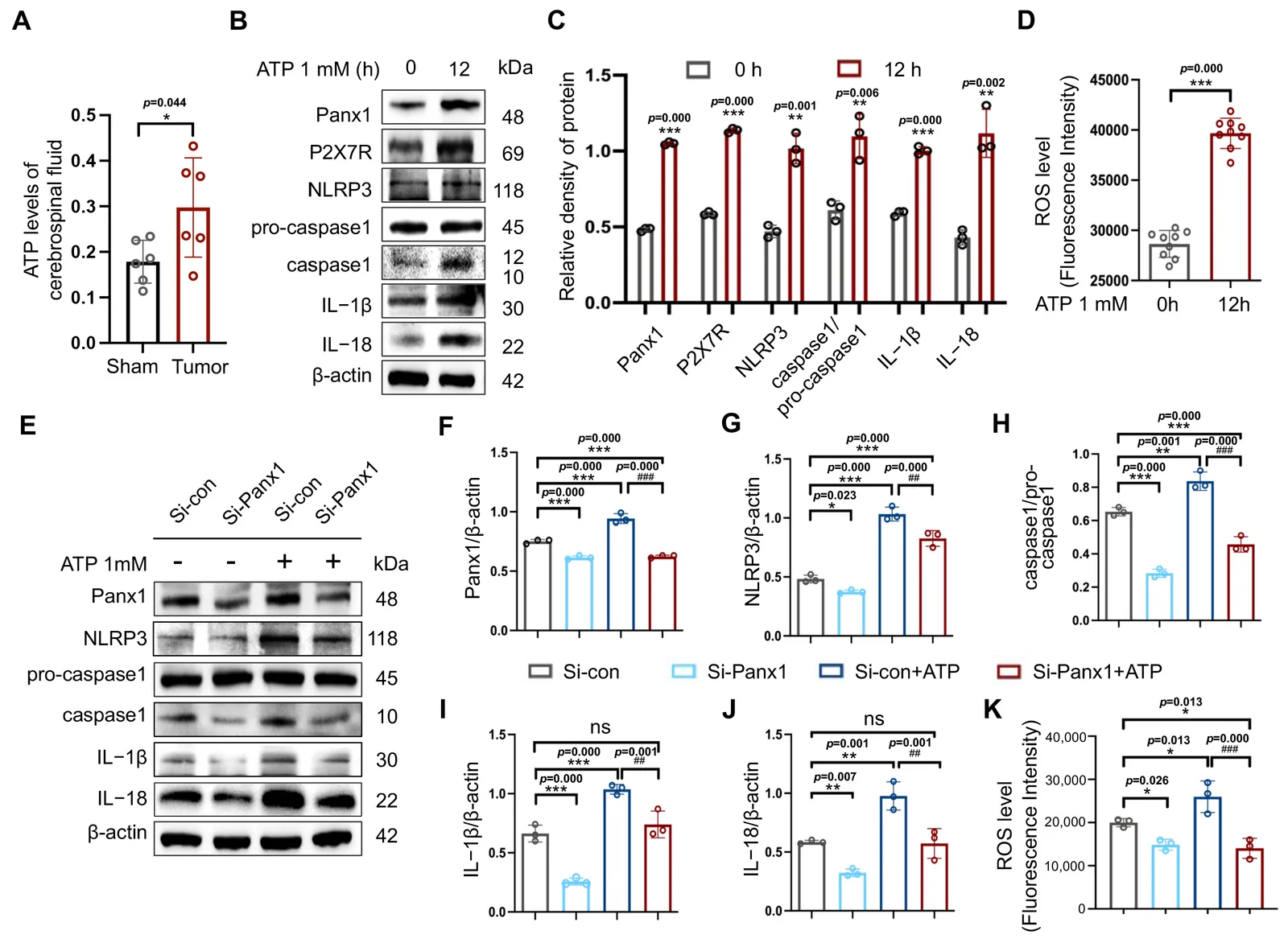
Panx1 is required for NLRP3 signaling activation-mediated releases of IL-1β and IL-18 following ATP treatment. (**A**) ATP levels were analyzed in the cerebrospinal fluid of Sham and Tumor mice on D21. *n* = 6/group (**B**,**C**) Western blotting of Panx1, P2X7R, NLRP3, pro-caspase1, caspase1, IL-18, and IL-1β (**B**), and their quantification analyses (**C**) in BV2 cells treated with 1 mM ATP. Comparisons between the two groups were performed by two-tailed unpaired *t*-tests. * 12 h vs. 0 h. (**D**) ROS levels in BV2 cells treated with 1 mM ATP. Comparisons between the two groups were performed by two-tailed unpaired *t*-tests. * 12 h vs. 0 h. (**E**–**J**) Western blotting of Panx1, NLRP3, pro-caspase1, caspase1, IL-18, and IL-1β (**E**) and their quantification analyses (**F**–**J**) in Si-con, Si-Panx1, Si-con + ATP, and Si-Panx1 + ATP group. Two-way ANOVA followed by the Bonferroni post hoc test was carried out to compare among groups. * vs. Si-con, ^#^ Si-con + ATP vs. Si-Panx1 + ATP. (**K**) ROS levels in Si-con, Si-Panx1, Si-con + ATP, and Si-Panx1 + ATP group. Two-way ANOVA followed by the Bonferroni post hoc test was carried out among groups. Panx1: F(3,8) = 118.125; NLRP3: F(3,8) = 122.223; caspase1/pro-caspase1: F(3,8) = 107.225; IL-1β: F(3,8) = 60.520; IL-18: F(3,8) = 27.556. * vs. Si-con, ^#^ Si-con + ATP vs. Si-Panx1 + ATP.

ATP is a key signaling molecule released in response to various nociceptive stimuli that critically modulates microglia activity. We found that ATP levels in the cerebrospinal fluid were significantly elevated in Tumor mice compared with those in the Sham group (Figure 6A). We therefore hypothesized that ATP was released in response to mechanical stimulation and further activates the NLRP3 through P2X7R-Panx1 signaling. Previous studies have shown that inhibition ATP signaling alleviates cancer-induced bone pain by suppressing ROS accumulation and inflammasome activation [28]. Here, ATP treatment markedly upregulated the expression levels of Panx1, P2X7R, NLRP3, caspase1, IL-1β, and IL-18 in BV2 cells (Figure 6B,C). In addition, ATP induced a two-fold elevation in ROS levels (Figure 6D). Importantly, ATP treatment did not affect cell viability (Supplementary Figure S1A), and flow cytometry with Annexin V/PI staining showed no significant increase in cell apoptosis (Supplementary Figure S1B,C), excluding the possibility that the elevated ROS levels and increased release of IL-1β and IL-18 were secondary to ATP-induced cell apoptosis. To further determine whether the NLRP3 was downstream of the P2X7R-Panx1 signaling, ATP-treated BV2 cells were transfected with Panx1 siRNA. Notably, Panx1 knockdown significantly inhibited the ATP-P2X7-NLRP3 axis and ROS accumulation (Figure 6E–K).

Collectively, these findings demonstrate that extracellular ATP may activate the Panx1–P2X7R signaling pathway in spinal microglia, thereby potentially promoting ROS accumulation, NLRP3 signaling activation, and production of the pro-inflammatory cytokines IL-1β and IL-18.

## 4. Discussion

Accumulating preclinical evidence indicates that microglia are broadly involved in both the induction and maintenance of hyperalgesia by regulating nociceptive signaling in the spinal cord [29]. Following bone tumor progression, bone destruction, and peripheral nociceptor sensitization, spinal activated microglia release numerous inflammatory mediators that enhance neuronal excitability and facilitate central sensitization [30]. However, the specific mechanisms by which microglia influence BCP remain largely unclear. Our previous studies demonstrated that persistent microglial activation contributes to BCP through multiple mechanisms. First, GABAergic synapses are pruned by activated microglia through suppression of CD47-SIRPα signaling in the SDH of Tumor group mice [31]. Second, pro-inflammatory cytokines released by activated microglia damage the blood–spinal cord barrier and exacerbate spinal neuroinflammation [32], allowing increased Th17 cell infiltration to further promote microglial activation during BCP development [33]. Third, a shift in microglial M1/M2 polarization toward the anti-inflammatory M2 phenotype could attenuate BCP [34]. The present study extends these observations by identifying Panx1-dependent purinergic signaling as an important molecular mechanism driving microglial inflammatory activation during BCP.

In the current study, BCP significantly upregulated IL-18 and IL-1β in parallel with a time-dependent increase in NLRP3 expression in the spinal cord. Upon activation, NLRP3 recruits and activates caspase1 through proteolytic cleavage of the p20 subunit, leading to the proteolytic maturation of IL-1β and IL-18. Both cytokines have been widely implicated in chronic pain by enhancing synaptic transmission and promoting central sensitization [27]. Consistent with these observations, our results showed that NLRP3 signaling activation exacerbated inflammatory responses through the caspase1/IL-18/IL-1β pathway, an established inflammatory axis. We interpret increased NLRP3 expression, combined with elevated caspase1 p20 cleavage and increased mature IL-1β and IL-18, as evidence of NLRP3 signaling activation rather than relying on NLRP3 expression alone. To unravel the mechanism by which NLRP3 activation promotes BCP, an NLRP3 inhibitor was applied in our model and significantly alleviated BCP. However, to establish the causal cellular source of NLRP3 activation contributing to BCP, future work should incorporate microglia-enriched isolation approaches or immunohistochemical co-localization analyses of NLRP3 with Iba1, GFAP, and NeuN.

Both priming and activation signals are indispensable for the activation of NLRP3 inflammasomes, which is commonly triggered by extracellular ATP acting on P2X7R, leading to K^+^ efflux, Ca^2+^ influx, and increased ROS levels [35]. Therefore, it is speculated that the P2X7R-Panx1 signaling may recognize the increased ATP activity and respond to induce NLRP3 expression upregulation. Components of the NLRP1 and NLRP2 inflammasome interact with P2X7R-Panx1 signaling in neurons and astrocytes, respectively [36]. Nevertheless, NLRP3, rather than the NLRP1 or NLRP2 inflammasome, is activated following Panx1 activation in microglia [37]. Despite well-documented roles of neuronal Panx1 in inflammatory pain and astrocytic Panx1 in neuropathic pain, no direct evidence has yet linked these cell-specific Panx1 pools to bone cancer pain progression [38]. Although Panx1-mediated ATP signaling has been implicated in neuropathic and inflammatory pain, its involvement in bone cancer pain remains insufficiently defined. This leaves open the question of whether neurons or astrocytes contribute to Panx1-mediated nociceptive signaling in BCP, which represents an important avenue for future investigation. In a neuropathic pain model induced by chronic constriction injury, increased NLRP3 expression is specific to microglia and astrocytes rather than neurons, whereas IL-1β release is observed only in microglia of the dorsal lumbar spinal cord [39]. In our study, immunofluorescence analysis confirmed that Panx1 and P2X7R were predominantly colocalized with the microglial marker Iba1 in the SDH of Tumor group mice. However, we acknowledge that the absence of NLRP3/Iba1 co-localization and the use of whole spinal cord tissue homogenates for Western blotting limit our ability to definitively attribute NLRP3 activation exclusively to microglia. Whole-tissue lysates include neuronal and astrocytic components, which may also contribute to the observed protein changes. Consistently, our findings demonstrated upregulation of P2X7R and Panx1 during BCP progression, accompanied by sustained activation of the NLRP3 signaling. The expression of Panx1, P2X7R, and NLRP3 increased over time, suggesting their involvement in BCP progression. However, these temporal changes reflect co-occurrence rather than establishing a sequential order of activation. More importantly, pharmacological inhibition of either Panx1 or P2X7R not only reduced pain behaviors but also suppressed NLRP3 signaling activation and cytokine production. These findings support the existence of a functional P2X7R-Panx1 signaling that amplifies spinal neuroinflammation during BCP. Nevertheless, our data only demonstrate temporal correlations rather than definitive sequential pathway activation.

Evidence from neurophysiological and pharmacological studies has demonstrated that ATP signaling on microglia can be attributed to an intertwined relationship between receptor-mediated signaling pathways and purinergic P2 receptors, offering multidimensional insights into the development and maintenance of pain [40]. In a female Wistar rats BCP model, bone cancer significantly increases ATP levels in the cerebrospinal fluid (CSF) and upregulates P2X7R and IL-18 in the spinal microglia [41]. Consistent with these findings, we observed significantly elevated ATP concentrations in the cerebrospinal fluid of tumor-bearing mice on day 21, suggesting enhanced purinergic signaling within the spinal cord. However, CSF ATP levels primarily reflect the net extracellular ATP pool and do not identify the cellular source of this increase. Previous studies have shown that dorsal horn neurons overexpressing the vesicular nucleotide transporter contribute to increased extracellular ATP content in the spinal cord after peripheral nerve injury [42]. In addition, extracellular ATP release from SDH astrocytes drives neuropathic mechanical allodynia through Panx1 hemichannels [43]. Beyond its role in ATP release, Panx1 appears to be a crucial component of a large protein-signaling platform bridging P2X7R to the components of the inflammasome, resulting in caspase1 activation and eventual releases of IL-1 family cytokines [44]. In particular, the release of IL-18 and IL-1β, which jointly participate in the modulation of pathological pain, can also be regulated by Panx1. In rats with joint injury-induced mechanical allodynia, it has been found that an increased release of spinal ATP and microglia-specific upregulation of Panx1 and P2X7R elicit subsequent Panx1-mediated IL-1β release [25]. Collectively, these findings suggest that elevated spinal ATP may act on Panx1 channels and thereby trigger a downstream cascade of inflammatory cytokine release, providing a potential mechanism linking enhanced purinergic signaling to the maintenance of BCP. In line with this hypothesis, we found that ATP stimulation induced robust activation of the Panx1–P2X7R–NLRP3 signaling cascade together with increased intracellular ROS production in cultured BV2 microglia, supporting a potential mechanism by which elevated spinal ATP in BCP may drive microglial inflammatory cytokine release. In addition, Panx1 knockdown markedly suppressed ATP-induced ROS accumulation, NLRP3 signaling activation, and pro-inflammatory cytokine production, indicating that Panx1 as a regulator of these inflammatory events. While these findings suggest that ATP acts, at least in part, through a P2X7R-Panx1-dependent pathway, we acknowledge that ATP is a promiscuous signaling molecule capable of activating multiple purinergic receptor subtypes. Therefore, we cannot exclude the possibility that other purinergic receptors contribute to the observed ATP-induced effects in our experimental system. Future studies using selective antagonists for other purinergic receptors or combinatorial knockdown approaches would be required to fully delineate the contribution of other ATP-sensitive pathways in microglial NLRP3 signaling activation in the context of BCP.

Previous studies have demonstrated that Panx1 channels, P2X7 receptors, and the NLRP3 inflammasome individually participate in neuroinflammatory responses under various pathological conditions, including neuropathic pain, inflammatory pain, and neurodegenerative diseases [17,25,37]. However, evidence directly linking these molecules into a coordinated signaling cascade that affects bone cancer pain remains limited. By combining behavioral assessments, pharmacological interventions, immunofluorescence analyses, and mechanistic experiments in ATP-stimulated microglia, our study provides complementary in vivo and in vitro evidence supporting the functional coupling of these signaling components during the development of bone cancer pain. We also found that increased extracellular ATP, most likely acting on microglia-Panx1 and inducing microglia activation, was involved in BCP progression. Panx1 knockdown in BV2 cells significantly reduced NLRP3 expression, and downregulated cleaved caspase1, IL-1β, and IL-18, indicating that Panx1 regulates these inflammatory events under these experimental conditions. However, we acknowledge that since exogenous ATP directly activates P2X7R, this experimental design partially bypasses the ATP-release function of Panx1. The observed effect of Panx1 knockdown likely reflects its role as a component of the P2X7R-associated signaling pathway that facilitates downstream inflammatory signaling, rather than its ATP channel function.

### Limitations

In the current study, a single BCP mouse model was established by inoculating NCTC 2472 murine fibrosarcoma cells into the femora of male C3He/J mice. This is a well-established and widely adopted model for investigating the mechanisms underlying bone cancer-induced mechanical hypersensitivity. To further validate the role of the Panx1/P2X7R/NLRP3 axis in regulating BCP, alternative models, such as Lewis lung carcinoma cell inoculation, could be employed in future studies. While our findings implicate microglial Panx1 as a regulator of P2X7R/NLRP3 signaling in bone cancer pain, certain limitations merit consideration. P2X7R is expressed in dorsal root ganglion satellite glial cells and is implicated in peripheral sensitization in visceral pain [45], but its role in bone cancer pain progression remains to be determined. This study was conducted exclusively in male C3He/J mice, as female mice were excluded to minimize confounding due to weight loss during the advanced stages of tumor progression. Furthermore, previous studies have reported no sex differences in bone cancer-induced pain hypersensitivity or spinal microglial responses, supporting a sex-independent mechanism of bone cancer pain in mice [18,46]. Nevertheless, our conclusions should be interpreted with caution when extrapolating to female subjects, and future studies incorporating both sexes are warranted to confirm the translational relevance of our findings. Rescue experiments are still required to conclusively establish the signaling hierarchy. Moreover, although microglia-specific Panx1 deletion has been validated in neuropathic and joint pain models, its absence in the bone cancer pain model leaves the cell-type specificity of the observed effects to be further corroborated [25]. Additionally, the lack of evidence for a direct physical interaction between Panx1 and P2X7R, together with the potential species-specific divergence in P2X7R function, warrants caution when extrapolation of our results to other experimental systems.

We acknowledge that the lack of direct clinical validation may limit the reliability of our mechanistic study. From a translational perspective, the present findings are of particular interest because all three components of this signaling pathway are pharmacologically targetable. Selective inhibitors of Panx1, P2X7R, and NLRP3 have shown encouraging anti-inflammatory and analgesic effects in preclinical studies of neurological and inflammatory disorders. ^10^Panx and BBG remain at the preclinical stage and are currently used primarily for mechanistic investigations. Probenecid, a clinically available broad-spectrum Panx1 blocker that is widely used to treat gout and hyperuricemia [47], has not yet been reported for BCP treatment. In contrast, NLRP3 inflammasome inhibitor MCC950 has entered a Phase II clinical trial for rheumatoid arthritis, although its application in pain management remains to be investigated [48].

## 5. Conclusions

Our study provides evidence that the Panx1/P2X7R/NLRP3 axis is associated with the development of BCP through inducing IL-18 and IL-1β releases in the SDH. Our findings also suggest that increased extracellular ATP may contribute to BCP progression, potentially via activation of microglial Panx1 and NLRP3 signaling. This is supported by our in vitro data showing that Panx1 knockdown in BV2 cells attenuated ATP-induced increased NLRP3 expression and reduced cleaved caspase-1, IL-1β, and IL-18 levels. However, further studies are needed to determine whether extracellular ATP acts primarily through this pathway in vivo and to dissect the relative contributions of other purinergic receptors in this process.

## Data Availability

The data generated in the present study may be requested from the corresponding author.

## Supplementary Materials

The following supporting information can be downloaded at: https://www.mdpi.com/article/10.3390/biomedicines14102183/s1, Figure S1: (A) Cell viability of BV2 cells treated with or without 1 mM ATP for 12 h, as assessed by CCK-8 assay. *n* = 3 independent experiments. ns, not significant (*p* = 0.5112). (B) Representative flow cytometry plots of BV2 cells stained with Annexin V-FITC and PI-PE after treatment with or without 1 mM ATP for 12 h. (C) Quantification of the percentage of PI^+^/Annexin V^+^ cells in CON and ATP-treated groups. *n* = 3 independent experiments. ns, not significant (*p* = 0.057); Table S1: Allocation of animals across all experimental groups and procedures.

## Abbreviations

The following abbreviations are used in this manuscript:

BCP	Bone cancer pain
SDH	Spinal dorsal horn
NLRP3	NOD-like receptor family pyrin domain containing 3
micro-CT	Micro-computed tomography
PWMT	Paw withdrawal mechanical threshold
NSF	Number of spontaneous flinches
ROS	Reactive oxygen species
CSF	Cerebrospinal fluid
