# Supplementary material for: Inhibition of Panx1/P2X7R/NLRP3 Signaling Attenuates Bone Cancer Pain and Spinal Microglial Activation
Source: Biomedicines. 2026 Sep 26;14(10):2183. doi: 10.3390/biomedicines14102183 (PMC13701744; doi:10.3390/biomedicines14102183)
Supplement: Supplementary file 1 [file biomedicines-14-02183-s001.zip › Supplementary Figure S1-figure legend.pdf]

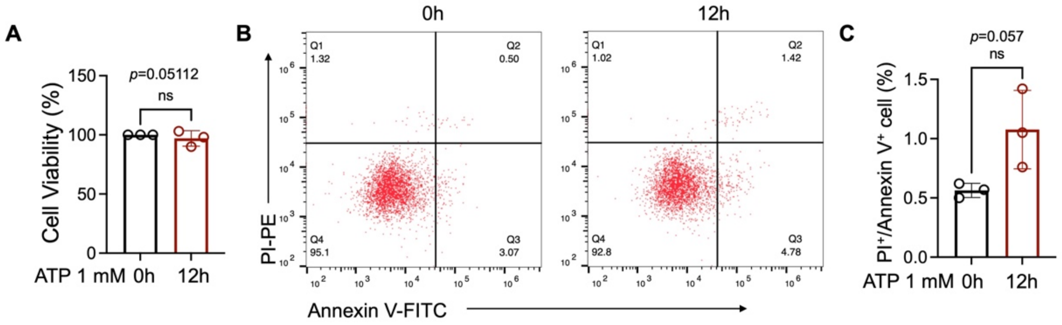

Supplementary Figure S1

(A) Cell viability of BV2 cells treated with or without 1 mM ATP for 12 h, as assessed by CCK-8 assay. *n* = 3 independent experiments. ns, not significant ($p = 0.5112$). (B) Representative flow cytometry plots of BV2 cells stained with Annexin V-FITC and PI-PE after treatment with or without 1 mM ATP for 12 h. (C) Quantification of the percentage of PI^+/Annexin V^+ cells in CON and ATP-treated groups. *n* = 3 independent experiments. ns, not significant ($p = 0.057$).
