# Supplementary material for: Inhibition of Panx1/P2X7R/NLRP3 Signaling Attenuates Bone Cancer Pain and Spinal Microglial Activation
Source: Biomedicines. 2026 Sep 26;14(10):2183. doi: 10.3390/biomedicines14102183 (PMC13701744; doi:10.3390/biomedicines14102183)
Supplement: Supplementary file 1 [file biomedicines-14-02183-s001.zip › Supplementary Table S1.pdf]

Experimental Groups	Experimental Procedures	Sample size (n)
Sham	PWMT (g)-Days after operation (d)	8
	NSF-Days after operation (d)	
	Western blotting (n=3 for indicated time points)	18
	IF	6
	HE staning	1
	Micro-CT	1
	CSF	6
Tumor	PWMT (g)-Days after operation (d)	8
	NSF-Days after operation (d)	
	Western blotting (n=3 for indicated time points)	18
	IF (n=6 D21)	6
	HE staning	1
	Micro-CT	1
	CSF	6
Sham+Vehicle(MCC950)	PWMT (g)-Time after injection	8
	NSF-Time after injection	
	Western blotting	3
Tumor+Vehicle(MCC950)	PWMT (g)-Time after injection	8
	NSF-Time after injection	
	Western blotting	3
Tumor+MCC950(10μg)	PWMT (g)-Time after injection	8
	NSF-Time after injection	
Tumor+MCC950(20μg)	PWMT (g)-Time after injection	8
	NSF-Time after injection	
Tumor+MCC950(40μg)	PWMT (g)-Time after injection	8
	NSF-Time after injection	
	Western blotting	3
Sham+Vehicle(BBG)	PWMT (g)-Time after injection	8
	NSF-Time after injection	
	Western blotting	3
Tumor+Vehicle(BBG)	PWMT (g)-Time after injection	8
	NSF-Time after injection	
	Western blotting	3
Tumor+BBG(0.03μg)	PWMT (g)-Time after injection	8
	NSF-Time after injection	
Tumor+BBG(0.06μg)	PWMT (g)-Time after injection	8
	NSF-Time after injection	
Tumor+BBG(0.012μg)	PWMT (g)-Time after injection	8
	NSF-Time after injection	
	Western blotting	3
Sham+Vehicle(10Panx)	PWMT (g)-Time after injection	8

	NSF-Time after injection	
	Western blotting	3
Tumor+Vehicle(10Panx)	PWMT (g)-Time after injection	8
	NSF-Time after injection	
	Western blotting	3
Tumor+10Panx(0.31μg)	PWMT (g)-Time after injection	8
	NSF-Time after injection	
Tumor+10Panx(0.62μg)	PWMT (g)-Time after injection	8
	NSF-Time after injection	
Tumor+10Panx(1.24μg)	PWMT (g)-Time after injection	8
	NSF-Time after injection	
	Western blotting	3
Total sample size (n)		227

Supplementary Table S1: Allocation of animals across all experimental groups and procedures.
